# Eculizumab in recalcitrant antiphospholipid antibody syndrome

**DOI:** 10.1186/ar4666

**Published:** 2014-09-18

**Authors:** Ekaterini Zapantis, Richard Furie, Diane Horowitz

**Affiliations:** 1 North Shore-Long Island Jewish Health System, Great Neck, NY, USA

## Background

Antiphospholipid syndrome (APS) is defined as the occurrence of venous or arterial thrombosis and/or pregnancy morbidity, in the presence of serological evidence of antiphospholipid antibodies (including IgM and IgG anticardiolipin antibodies, IgM and IgG anti-β_2_-glyoprotein I antibodies, or the lupus anticoagulant). Whereas most patients with focal thrombotic events respond to anticoagulation, occasional patients are refractory to standard therapeutic interventions and continue to have either focal or multifocal occlusive disease. For those with recalcitrant disease or those with the catastrophic antiphospholipid syndrome (CAPS), physicians resort to the addition of antiplatelet agents, steroids, immunosuppressives, IVIG, rituximab, or plasma exchange.

Complement inhibition may be an effective way to prevent thrombosis associated with APS. Eculizumab, a monoclonal antibody that binds to complement protein C5 and prevents the conversion of C5 to C5a and C5b, may potentially be an effective treatment for patients with APS. First studied in patients with systemic lupus erythematosus, rheumatoid arthritis, dermatomyositis, and idiopathic membranous nephropathy in the early 2000s, development of the drug for rheumatic diseases was abandoned in favor of paroxysmal nocturnal hemoglobinuria and atypical hemolytic uremic syndrome. Given the experience of complement inhibition in animal models of APS as well as prior use of eculizumab several years ago in one of our refractory APS patients, we administered eculizumab to two patients with severe refractory APS.

## Methods

Two patients with antiphospholipid syndrome, unresponsive to conventional anticoagulant therapy, were treated with a loading dose of eculizumab followed by dosing every other week (atypical hemolytic uremic syndrome dosing schedule). It has been suggested that the platelet count may be used as a surrogate marker of APS activity. During therapy, both patients' platelet counts were monitored and any new thrombotic events documented. At the time of submission of this abstract, both patients are continuing treatment with eculizumab.

## Results

**Figure 1:**
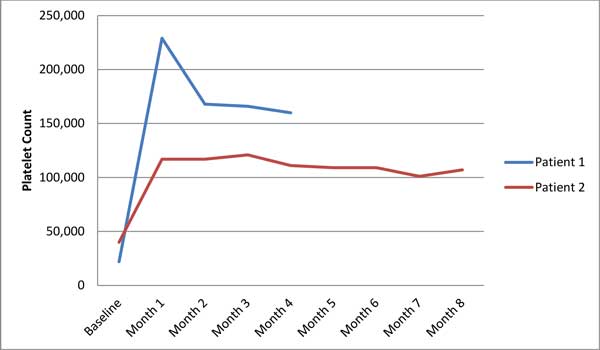
**APS patients treated with eculizumab: platelet counts**.

At their lowest values, the patients had platelet counts of 35,000 and 22,000 (K/ml). One of the patients was steroid dependent in order to maintain her platelet count. After initiation of eculizumab, the patient was able to taper steroids as the platelet count has risen from a low of 35,000 to average counts of 100,000. The second patient's platelet count rose to over 200,000 from 22,000 within 10 days of receipt of eculizumab. For both patients the increases in platelet counts have been sustained other than during brief periods when therapy was delayed. During the treatment period (4 and 8 months), there were no new thrombotic events. See Figure 1.

## Conclusions

Eculizumab has shown promising results in our patients with refractory antiphospholipid syndrome. Longer follow-up of these patients will be needed in order to discern the effect on thrombosis. Controlled studies are needed to further assess the efficacy of eculizumab in this condition, as are mechanistic studies.

